# Methadone in combination with magnesium, ketamine, lidocaine, and dexmedetomidine improves postoperative outcomes after coronary artery bypass grafting: an observational multicentre study

**DOI:** 10.1186/s13019-024-02935-0

**Published:** 2024-06-26

**Authors:** Laurence Weinberg, Samuel Johnston, Luke Fletcher, Rebecca Caragata, Riley H. Hazard, Peter Le, Jadon Karp, Bradly Carp, Sui Wah Sean Yip, Dominic Walpole, Nicholas Shearer, Tom Neal-Williams, Robert Nicolae, Angelica Armellini, George Matalanis, Siven Seevanayagam, Rinaldo Bellomo, Timothy Makar, Param Pillai, Stephen Warrillow, Ziauddin Ansari, Anoop N. Koshy, Dong-Kyu Lee, Michael Yii

**Affiliations:** 1 Department of Anesthesia, Austin Health, Heidelberg, Australia; 2 Department of Cardiac Surgery, Epworth Eastern Hospital, Melbourne, Australia; 3 Department of Cardiac Surgery, Austin Hospital, Melbourne, Australia; 4 Department of Intensive Care, Austin Health, Melbourne, Australia; 5 Department of Intensive Care, Epworth Eastern Hospital, Melbourne, Australia; 6 Department of Cardiology, Austin Health, Melbourne, Australia; 7 Department of Anesthesiology and Pain Medicine, Dongguk University Ilsan Hospital, Goyang, Republic of Korea

**Keywords:** Cardiac surgery, Anesthesia, Analgesia, Methadone, Dexmedetomidine, Lidocaine, Magnesium, Ketamine

## Abstract

**Background:**

An optimal pharmacological strategy for fast-track cardiac anesthesia (FTCA) is unclear. This study evaluated the effectiveness and safety of an FTCA program using methadone and non-opioid adjuvant infusions (magnesium, ketamine, lidocaine, and dexmedetomidine) in patients undergoing coronary artery bypass grafting.

**Methods:**

This retrospective, multicenter observational study was conducted across private and public teaching sectors. We studied patients managed by a fast-track protocol or via usual care according to clinician preference. The primary outcome was the total mechanical ventilation time in hours adjusted for hospital, body mass index, category of surgical urgency, cardiopulmonary bypass time and EuroSCORE II. Secondary outcomes included successful extubation within four postoperative hours, postoperative pain scores, postoperative opioid requirements, and the development of postoperative complications.

**Results:**

We included 87 patients in the fast-track group and 88 patients in the usual care group. Fast-track patients had a 35% reduction in total ventilation hours compared with usual care patients (*p* = 0.007). Thirty-five (40.2%) fast-track patients were extubated within four hours compared to 10 (11.4%) usual-care patients (odds ratio: 5.2 [95% CI: 2.39–11.08; *p* < 0.001]). Over 24 h, fast-track patients had less severe pain (*p* < 0.001) and required less intravenous morphine equivalent (22.00 mg [15.75:32.50] vs. 38.75 mg [20.50:81.75]; *p* < 0.001). There were no significant differences observed in postoperative complications or length of hospital stay between the groups.

**Conclusion:**

Implementing an FTCA protocol using methadone, dexmedetomidine, magnesium, ketamine, lignocaine, and remifentanil together with protocolized weaning from a mechanical ventilation protocol is associated with significantly reduced time to tracheal extubation, improved postoperative analgesia, and reduced opioid use without any adverse safety events. A prospective randomized trial is warranted to further investigate the combined effects of these medications in reducing complications and length of stay in FTCA.

**Trials registration:**

The study protocol was registered in the Australian New Zealand Clinical Trials Registry (https://www.anzctr.org.au/ACTRN12623000060640.aspx, retrospectively registered on 17/01/2023).

**Supplementary Information:**

The online version contains supplementary material available at 10.1186/s13019-024-02935-0.

## Background

More than 800,000 patients undergo coronary artery bypass graft (CABG) each year, making it the most performed cardiac surgery worldwide [1]. Variation in the quality of perioperative care has prompted the establishment of quality measures through the Society of Thoracic Surgeons and National Quality Forum [2, 3]. Despite these endeavors, little effort has been directed toward optimizing or standardizing postoperative care after CABG. Up to 36% of cardiac surgery patients have a prolonged length of stay (LOS) in the intensive care unit (ICU), [4] which results in higher health costs. The increasing demand for cardiac surgery has prompted clinicians to explore improved strategies for safe and effective recovery models to enhance patient outcomes while optimizing resource utilization.

Contemporary fast-track cardiac anesthesia (FTCA) aims for tracheal extubation to occur within four hours post-surgery and discharge from the ICU within 24 h [5]. FTCA programs minimize variability in care and improve efficient use of resources without compromising clinical efficacy or patient safety outcomes [6–10]. The optimal pharmacological strategy for FTCA has not been established. Methadone, dexmedetomidine, lidocaine, magnesium, and ketamine have been reported to be beneficial in the enhanced recovery of patients undergoing major surgery; however, their combination to facilitate FTCA has not been investigated.

This study aimed to evaluate the effectiveness and safety of an FTCA program using methadone with non-opioid adjuvant infusions (magnesium, ketamine, lidocaine, and dexmedetomidine) in patients undergoing CABG. We hypothesized that successful and safe fast-track CABG can be achieved in selected candidates using this approach.

## Methods

This retrospective multicenter observational study was conducted at two hospitals in Victoria, Australia. Austin Hospital is a quaternary referral public hospital specializing in high-risk cardiac surgery that performs approximately 540 open cardiac procedures annually. Epworth Eastern is a private university teaching hospital undertaking complex cardiac surgery that performs approximately 200 open cardiac procedures annually. Both hospitals are served by cardiologists, cardiac surgeons, anesthesiologists, and intensivists working across both health facilities. Accordingly, the patients at each hospital were managed using the same cardiac-anesthesia protocols and guidelines. The Austin Health Human Research Ethics Committee approved this study and waived the requirement for participant consent (approval number 22/Austin/38; approval date 24/03/2022). The study protocol was registered in the Australian New Zealand Clinical Trials Registry (https://www.anzctr.org.au/ACTRN12623000060640.aspx, retrospectively registered on 17/01/2023) [11]. The study was reported following the Strengthening the Reporting of Observational Studies in Epidemiology guidelines [12].

### Inclusion and exclusion criteria

A FTCA program was implemented in our institution in 2019 after a quality improvement program demonstrated its safety and feasibility [13]. However, outcomes from the FTCA program have never been compared to patients undergoing cardiac surgery using standard of care practices. Therefore, patients who underwent primary CABG surgery via midline sternotomy were screened between February 2019 and February 2022. Patients managed by a fast-track protocol (fast-track group) or usual care (usual care group) were included. Given that this is a retrospective observational study, there was no random assignment of participants to the different groups, and the two groups above occurred naturally based on exposure to FTCA or no exposure.

Exclusion criteria included those undergoing time-critical salvage CABG (e.g., patients admitted with out-of-hospital arrest in cardiogenic shock requiring emergency CABG), patients undergoing CABG combined with valve surgery, surgery on the aorta, and redo surgeries. In addition, patients with a preoperative cardiac assist device in situ and those who underwent CABG after 6 p.m. were excluded, as these patients often remained on mechanical ventilation support until the next postoperative morning.

### Fast-track care and usual care

The patients received standard surgical care from the attending cardiac surgeon. Routine monitoring included a single brachial or femoral arterial line, a pulmonary artery catheter, and intraoperative transesophageal echocardiography. Continuous central venous and pulmonary artery pressures were monitored from the proximal and distal ports of the catheter.

**Fig. 1:**
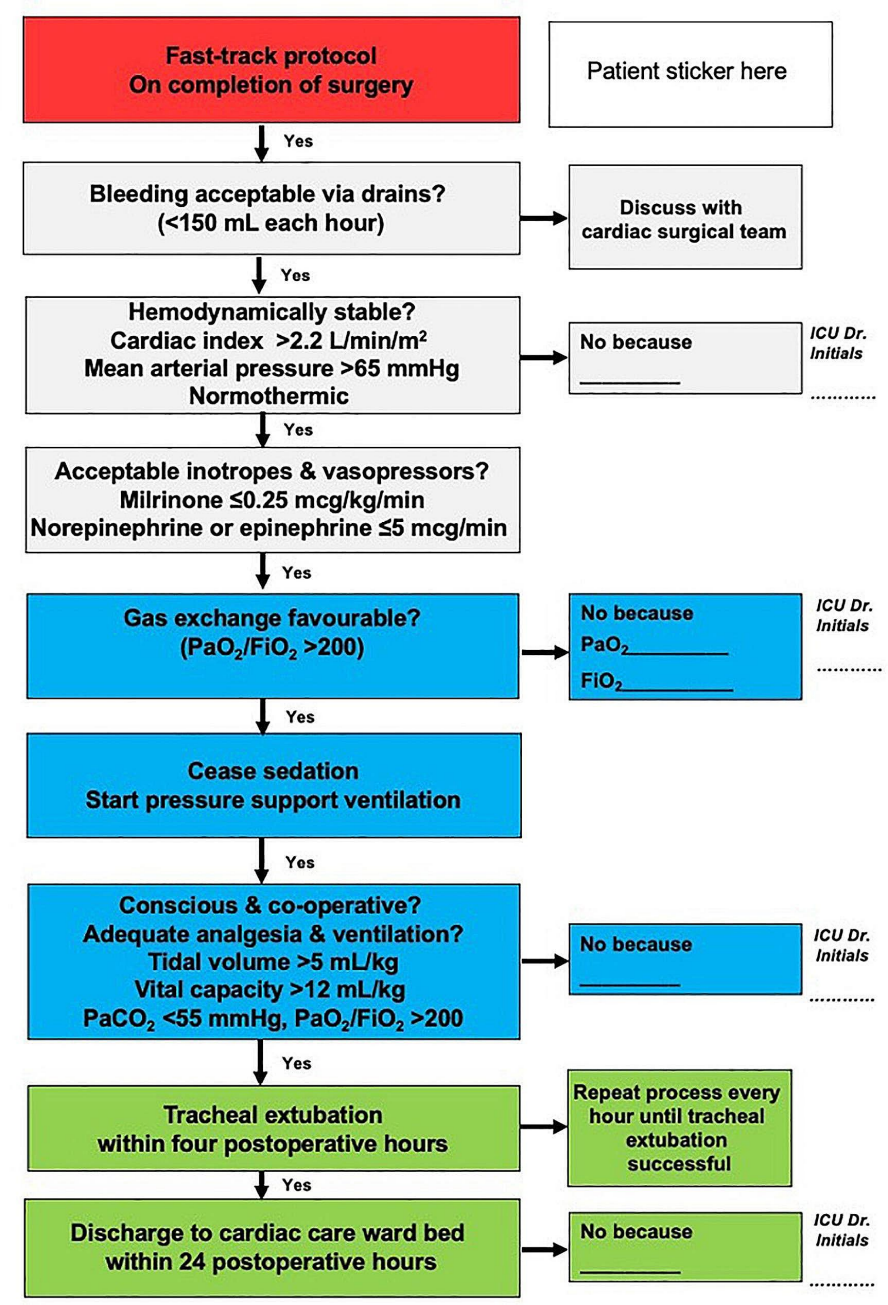
Postoperative mechanical ventilation protocol for all patients

Additionally, all patients received processed electroencephalography monitoring using the bispectral index (BIS™ Quatro Brain Monitoring Sensor) or patient state index (PSI; Sedline, Masimo, Irvine, CA, USA). Monitoring was commenced prior to induction of anesthesia and the PSI and BIS were maintained at 25–50 and 40–60 respectively, during induction of anesthesia, during cardiopulmonary bypass, and throughout the duration of surgery. Central venous catheters were used for the infusion of vasoactive medications and other infusions, and cerebral oximetry was performed at the discretion of the attending anesthesiologist. The anesthesiologist or intensivist used the same ventilation weaning and tracheal extubation protocols for all patients (see Fig. 1). The perioperative protocols for the Fast-track and Usual care groups are summarized in Table 1.

In the Usual Care group, anesthesiologists selected muscle relaxants and reversal agents according to their individual clinical preferences and experience rather than following a standardized protocol. This approach allowed them to tailor the drug choice to each patient’s unique needs and surgical context, taking into account factors such as patient health status, type and duration of surgery, and potential drug interactions. Such individualized decision-making reflected the diverse practices and therapeutic strategies employed by anesthesiologists caring for patients in the Usual care group. All patients in the FTCA group received a patient-controlled analgesic device to empower autonomy over their pain management and facilitate consistent and immediate pain relief.

**Table 1:** Perioperative protocol for fast-track cardiac anesthesia and usual care. All drug doses were calculated as “actual body weight” unless otherwise stated

Fast-track group	Usual care group
**Preoperatively**	**Preoperatively**
Routine preoperative cardiac workup and optimization	Routine preoperative cardiac workup and optimization
**Preoperative medication (at the time of vascular access insertion in theatre)**	**Preoperative medication (1 h preoperatively or at the time of vascular access insertion in theatre)**
Methadone (0.1–0.2 mg/kg IV) Bolus aliquots of propofol (10–20 mg) for sedation	Temazepam (10 mg per os) and/or opioid (oxycodone [10 mg per os] or morphine [10 mg SC])
**Induction of anesthesia guided by the processed electroencephalogram to ensureadequate monitoring for depth of anesthesia**	**Induction of anesthesia guided by the processed electroencephalogram to ensure adequate monitoring for depth of anesthesia**
Remifentanil (1–2 µg/kg IV) Propofol (10–50 mg IV) Neuromuscular blockers: rocuronium (1 mg/kg) or vecuronium (0.1 mg/kg)	Fentanyl (5–10 ug/kg IV) Propofol (10–50 mg IV) Neuromuscular blocking agent: at the discretion of the treating anesthesiologist i.e., pancuronium, vecuronium, rocuronium or cisatracurium tailored to each patient’s unique needs and surgical context.
**Maintenance anesthesia including cardiopulmonary bypass**	**Maintenance anesthesia including cardiopulmonary bypass**
Dexmedetomidine load (0.5 µg/kg IV over 30 min) Dexmedetomidine infusion (0.5 µg/kg/h IV) Remifentanil infusion (0.1–0.3 µg/kg/min IV) or target control infusion (3–6 ng/mL IV) Lidocaine (0.5 mg/kg/h IV) (ideal body weight dosing) Magnesium (10 mg/kg/h IV) Propofol target control infusion (1–3 µg/mL IV) or volatile anesthesia to maintain < 50 PSI, < 60 BIS or to control blood pressure	Fentanyl boluses (100–500 µg IV) Propofol target control infusion (1–3 µg/mL IV), volatile anesthesia to maintain 25–50 PSI, 40–60 BIS bypass circuit, or to reduce control blood pressure if needed
**Fluids, vasoactive medications, and blood products**	**Fluids, vasoactive medications, and blood products**
At the discretion of the anesthesiologists guided by clinical context, echocardiographic and pulmonary artery assessments, and blood loss.	At the discretion of the anesthesiologists guided by clinical context, echocardiographic and pulmonary artery assessments, and blood loss.
**Post CPB**	**Sternal closure**
Ketamine (0.05–0.1 mg/kg/h) Paracetamol (1 g)	Fentanyl (100–500 µg IV) or oxycodone (5–10 mg) boluses
**Skin closure**	**Skin closure**
All infusions stopped except ketamine Single pass oral gastric tube to decompress stomach and suction any gastric contents, then gastric tube to be removed	Nasogastric or oral gastric tube left in situ to decompress stomach and suction any gastric contents
**Completion of skin closure**	**Completion of skin closure**
Sugammadex for reversal of neuromuscular blockade (400 mg) Anesthesia agents stopped and weaning from mechanical ventilation protocol (see Fig. 1) Propofol infusion commenced at 100–200 mg/hr if transferred to ICU	Propofol infusion commenced at 100–200 mg/hr Reversal of neuromuscular blocking agent at the discretion of the anesthesiologist
**Postoperative analgesia**	**Postoperative analgesia**
Ketamine (0.05–0.1 mg/kg/h) Patient-controlled analgesia fentanyl (10 µg/bolus, 5 min boluses, 5 min lockout, no background infusion) Paracetamol (1 g IV) every 6 h for 48 h	Nurse or clinician-directed fentanyl (20 ug – 40ug) or morphine (1–2 mg) boluses Paracetamol (1 g IV) every 6 h for 48 h
**Postoperative agitation/delirium**	**Postoperative agitation/delirium**
**Non-pharmacological interventions** Reorientation and cognitive stimulation with clocks, calendars, and familiar objects from home. Environmental modifications include adequate lighting, reduced noise, and adequate sleep hygiene. **Pharmacological interventions** First line: Quetiapine orally or via a nasogastric tube (12.5–25 mg BD daily) and titrate if needed to a daily dose 50 mg BD or Olanzapine (2.5–5 mg) sublingual or via a nasogastric tube daily. Second line: dexmedetomidine IV (0.3–0.8 ug/kg/hr)	**Non-pharmacological interventions** Reorientation and cognitive stimulation with clocks, calendars, and familiar objects from home. Environmental modifications include adequate lighting, reduced noise, and adequate sleep hygiene. **Pharmacological interventions** First line: Quetiapine orally or via a nasogastric tube (12.5–25 mg BD daily) and titrate if needed to a daily dose 50 mg BD or Olanzapine (2.5–5 mg) sublingual or via a nasogastric tube daily. Second line: dexmedetomidine IV (0.3–0.8 ug/kg/hr)

### Standardization of cardiopulmonary bypass

All patients received a 1500 ml pump prime containing PlasmaLyte 148. The circuit was primed with 10,000 IU of heparin. Cardiopulmonary bypass (CPB) was performed using a membrane oxygenator (Quadrox-I, Maquet Cardiopulmonary, Hirrlingen, Germany) with a pump rate of 2.2–2.4 L/m^2^/min. The mean arterial pressure (MAP) was maintained at 60–80 mmHg, and oxygen delivery was standardized at > 272 ml O_2_/kg/min. The hemoglobin level was maintained at > 70 g/dl. A target body temperature of 33–34 °C was maintained in all patients. All patients underwent standard induction cardioplegia (anterograde 600 mL followed by retrograde 400 mL delivered at 20 °C). The total arresting dose was approximately 13 ml/kg, 20 m equivalents of KCI. The maintenance dose was retrograde cardioplegia (400–500 mL), delivered at 20 °C every 15 min.

### Predefined outcome variables

The primary outcome was the total mechanical ventilation time (in hours), which was adjusted for several factors associated with prolonged postoperative mechanical ventilation times and increased length of ICU stay, including the hospital, body mass index, category of surgical urgency, cardiopulmonary bypass (CPB), and European System for Cardiac Operative Risk Evaluation (EuroSCORE II) [14–17].

Secondary outcomes included successful extubation within four postoperative hours, time taken to tracheal extubation, pain within the first 24 h postoperatively, total intravenous (IV) opioid requirements within the first 24 and 48 postoperative hours, and time to mobilization in the ICU in hours. The pain score was based on a numerical rating scale, where 0 indicated no pain and 10 indicated the worst pain experienced. Severe pain was defined as a pain score > 6. Other outcomes included the development of respiratory depression requiring naloxone, need for non-invasive respiratory support, failed extubation, and development of pneumonia. Sedation was measured with the Richmond Agitation-Sedation Scale [18].

Complications included delirium, acute kidney injury (AKI), bleeding or requirement for blood transfusion, cardiac arrhythmias, need for a permanent pacemaker, cerebrovascular events, and surgical site infection. Complications were defined by the European Perioperative Clinical Outcome definitions [19] (see Table 1: Definitions of complications, in the Supplementary File 1). Data on intensive care unit (ICU) and hospital length of stay (LOS), readmissions within 30 postoperative days, and in-hospital and 30-day mortality were also collected.

### Data collection

Preoperative data were extracted from the electronic medical records of each hospital by four investigators. A fifth investigator checked the data metric differences by re-interrogating the medical records. Preoperative data included patient demographics, anthropometric measurements, hematological and biochemical blood test results, echocardiography results, and comorbidities.

Intraoperative data collected included the type and dose of anesthetic drugs administered, CPB and aortic clamp times, administration of blood products, arterial blood gas results, and duration of surgery. Postoperative data included time to tracheal extubation, sedation scores, requirements for vasoactive drugs, type and volume of intravenous fluids, complications, ICU and hospital LOS, readmissions, and mortality within 30 postoperative days.

### Statistical analysis

Statistical analysis was performed using R 4.2.0 (R Development Core Team, Vienna, Austria, 2022) and associated packages [20] (see Table 2: R-packages, in the Supplementary File 2). Normality was tested by graphical methods using a quantile-quantile plot for continuous variables. The patient characteristics and postoperative outcome associations between Fast-track and Usual care groups were investigated using the Wilcoxon-Mann-Whitney test for continuous variables and Fisher’s exact or chi-squared test for categorical variables.

Violin plots were constructed to compare the data distribution of the unadjusted values of total mechanical ventilation time between the groups. The Wilcoxon-Mann-Whitney test was used to test for statistical significance between the two violin plots. To investigate the adjusted difference in ventilation time between the fast track and usual care groups, a linear regression model was built. We examined the estimated difference in total ventilation hours among patients who received postoperative sedation and ventilator care. Logarithmic transformation of the total mechanical ventilation time was done to improve the normality of the data and to reduce the impact of outliers. Allocation to either the fast-track or the usual care group was used as an independent variable. Body mass index, category of surgery, whether the surgery was performed in a public or private hospital, CPB time, and EuroSCORE II were the a priori selected covariates.

A modified survival plot was created to model “time-to-event,” where time was recorded in hours and the event was defined as tracheal extubation. The Kaplan-Meier model was then used to compare the differences between the fast-track and usual care groups. The log-rank test was used to calculate the statistical significance between the two groups in the survival plot.

Box plots were used to compare the secondary outcomes of total equivalent IV morphine use in milligrams between the groups in the 0–24-hour and 24–48-hour periods. The Wilcoxon-Mann-Whitney test was used to calculate the statistical significance between the Fast-track and Usual care groups during these periods. Data are expressed as the median (1st:3rd quartile) or number (percentile). All the calculated *p*-values were two-sided. Statistical significance was set at a *p*-value of 0.05. The complete deidentified dataset is available in the Supplementary File 3.

## Results

**Fig. 2:**
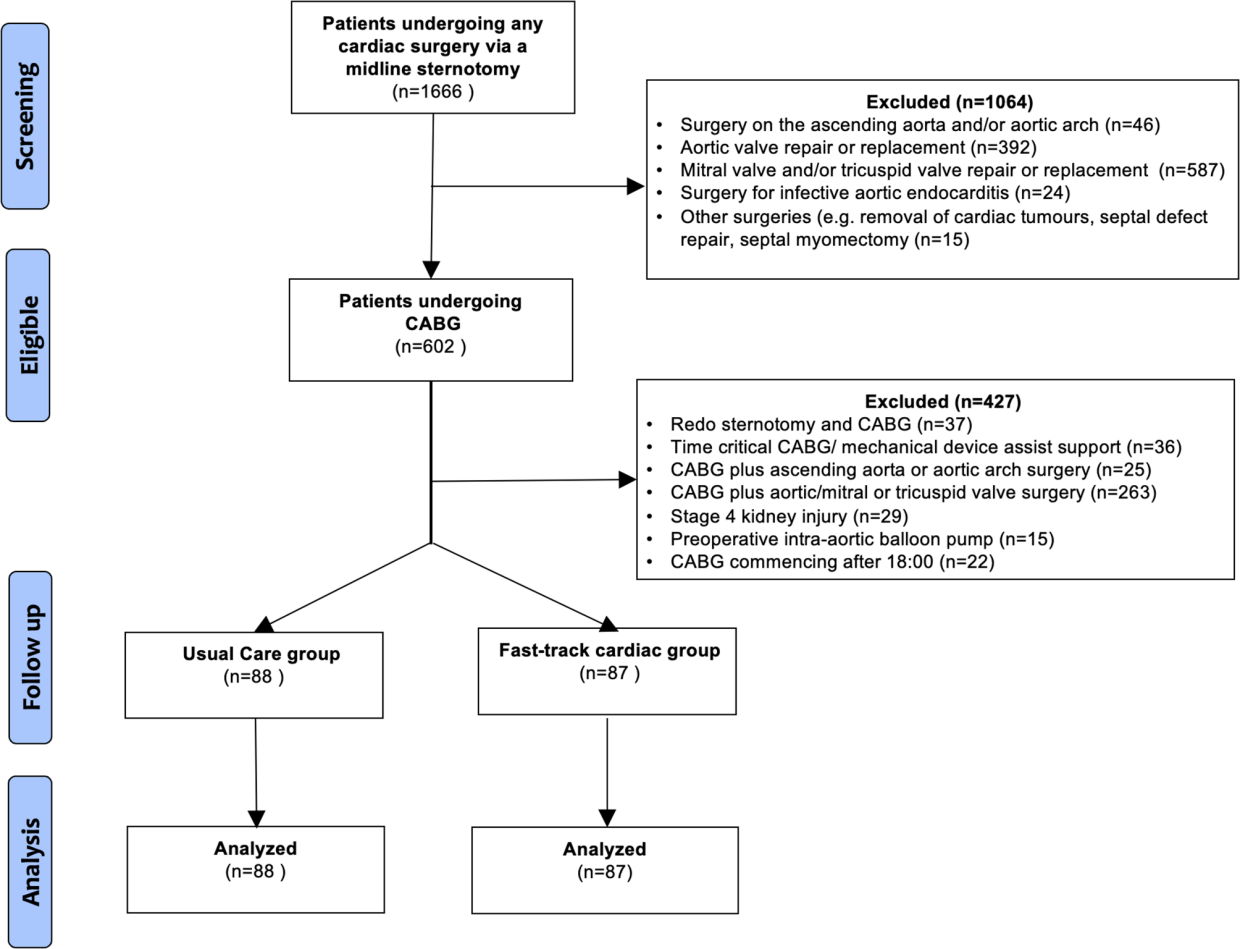
Flow diagram

During the study period, 1666 patients underwent cardiac surgery requiring midline sternotomy and CPB. The numbers of patients excluded are summarized in the study diagram (see Fig. 2). One hundred and seventy-five patients fulfilled the inclusion criteria: 87 patients in the fast-track group and 88 patients in the usual care group. In total 62/87 (71%) fast-track patients and 33/88 (38%) of the usual care patients were treated in a private hospital.

Baseline patient characteristics and differences in preoperative variables are presented in Table 2. No significant differences in overall baseline characteristics including age, gender, BMI and surgical risk as per the EUROSCORE-II were noted between the Fast-track and Usual Care groups. However, patients in the fast-track group were less likely to have diabetes mellitus and chronic kidney disease, and were more likely to be of Caucasian ethnicity, and non-smokers.

**Table 2:** Preoperative characteristics. Data are presented as a number (proportion) or a median (interquartile range)

Preoperative characteristic	Fast-track (*n* = 87)	Usual care (*n* = 88)	*P*-value
Proportion of all patients	49.71%	50.29%	N/A
Age (years)	70.00 [62.00:74.50]	71.50 [61.75:76.00]	0.623
Male gender	74 (85.10%)	75 (85.20%)	> 0.999
Body mass index (kg/m^2^)	27.70 [25.90:32.10]	29.2 [26.20:31.75]	0.309
**Triage category**			
Elective	60 (69.00%)	66 (75.00%)	0.403
Urgent	5 (5.75%)	14 (15.90%)	0.030
Emergent	22 (25.30%)	8 (9.09%)	0.005
Urgent and emergent combined	27 (31.0%)	22 (25%)	0.404
EuroSCORE II (%)	1.18 [0.75:1.96]	1.02 [0.78:1.48]	0.315
**Ethnicity**			
Caucasian	77 (88.50%)	64 (72.70%)	
Torres Strait Islander	3 (3.45%)	22 (25.00%)	
Asian Indian Indigenous Missing data	5 (5.75%) 1 (1.15%) 1 (1.15%) 0 (0.00%)	0 (0.00%) 0 (0.00%) 1 (1.14%) 1 (1.14%)	< 0.001
**Comorbidities**			
Acute myocardial infarction	4 (4.60%)	8 (9.09%)	0.371
Congestive cardiac failure	1 (1.15%)	2 (2.27%)	> 0.999
Peripheral vascular disease	0 (0.00%)	1 (1.14%)	> 0.999
Chronic pulmonary disease	0 (0.00%)	3 (3.41%)	0.246
Rheumatoid disease	0 (0.00%)	3 (3.41%)	0.246
History of liver disease	0 (0.00%)	1 (1.14%)	> 0.999
Transient ischemic attacks	0 (0.00%)	1 (1.14%)	> 0.999
Chronic kidney disease	0 (0.00%)	10 (11.40%)	0.001
Diabetes mellitus (with or without complications)	7 (8.05%)	31 (35.20%)	< 0.001
Cancer history	1 (1.15%)	3 (3.41%)	0.621
Hypertension	68 (78.2%)	67 (76.10%)	0.857
Cerebral vascular disease	4 (4.60%)	8 (9.09%)	0.371
Smoking status			
Non-smoker Ex-smoker Smoker	61 (70.10%) 11 (12.60%) 15 (17.20%)	29 (33.00%) 13 (14.80%) 46 (52.30%)	< 0.001
**Preoperative bloods**			
Hemoglobin (g/L) Platelets (x10^9^) Creatinine (*u*mol/L) Estimated glomerular filtration rate (mL/min/1.73 m^2^) Albumin (g/L) Hemoglobin A1c (%) Ferritin (*u*gL) Prothrombin time (sec) Activated partial thromboplastin time (sec)	139.00 [127.00:148.00] 228.00 [197.00:281.00] 88.00 [75.50:101.00] 90.00 [78.50:90:00] 36.00 [33.50:40.00] 6.40 [5.43:7.92] 123.00 [54.00:125:00] 12.00 [12.00:13.00] 29.00 [25.20:32.00]	142.00 [126.00:151.00] 226:00 [186.00:270.00] 84.00 [72.50:100.00] 78.50 [61.00:90:00] 39.00 [37.00:41.00] 6.10 [5.40:7.20] 149.00 [71.20:289.00] 12.00 [11.00:14.00] 30.00 [27.00:33.00]	0.578 0.206 0.745 < 0.001 < 0.001 0.533 0.315 0.609 0.070
**Pulmonary artery pressures**			
Normal (< 20 mmHg)	74 (85.10%)	50 (56.80%)	
Mild (20–40 mmHg) Moderate (> 41–55 mmHg) Not reported	1 (1.15%) 0 (0.00%) 12 (10.20%)	9 (10.20%) 3 (3.41%) 26 (29.50%)	0.001
**Left ventricular ejection fraction (%)**	56.00 [50.00:62.80]	60.00 [52.00:65:00]	0.057
**Regional wall function**			
Normal Regional wall abnormalities Mild or moderate systolic dysfunction Severe dysfunction	65 (74.70%) 19 (21.80%) 3 (3.45%) 0 (0.00%)	56 (63.60%) 24 (27.30%) 7 (7.95%) 1 (1.14%)	0.241
**Right ventricle dilated**	5 (5.75%)	7 (7.95%)	0.766
**Right ventricle impairment**	2 (2.30%)	3 (3.41%)	> 0.999

Patients in the private sector had a higher median EUROSCORE II: 1.46 (0.87:2.55) vs. 0.95 (0.74:1.22); *p* < 0.001). The intraoperative data are presented in Table 3. Patients in the fast-track group had significantly longer median aortic clamp times: 112.00 min (90.00:138.00) vs. 80.00 min [65.80:96.20]; *p* < 0.001 and longer median CPB times: 133.00 min (110.00:156:00) vs. 101.00 min (88.00:124.00); *p* < 0.001, compared to the usual care group.

**Table 3:** Intraoperative data. Data are presented as a number (proportion) or a median (interquartile range)

		Fast-track (*n* = 87)	Usual care (*n* = 88)	*P*-value
Premedication		0 (0.00%)	88 (100.00%)	N/A
Premedication type	Midazolam Diazepam Temazepam	0 (0.00%) 0 (0.00%) 0 (0.00%)	76 (86.40%) 39 (44.30%) 17 (19.30%)	N/A
**Neuromuscular blocking agent**				
Rocuronium	Patients receiving	87 (100.0%)	26 (29.5418%)	< 0.0001
Vecuronium	Patients receiving	0 (0.00%)	18 (20.45%)	N/A
Pancuronium	Patients receiving	0 (0.00%)	39 (44.31%)	N/A
Cisatracurium	Patients receiving	0 (0.00%)	10 (11.36%)	N/A
**Neuromuscular reversal agent at end of case**				
Sugammadex	Patients receiving	87 (100.0%)	14 (15.91.%)	0.001
Glycopyrrolate/neostigmine	Patients receiving	0 (0.00%)	21 (23.86%)	N/A
No reversal agent administered	Patients receiving	0 (0.00%)	53 (60.22%)	N/A
**Opioid use**				
Fentanyl	Patients receiving Median dose (*µ*g)	0 (0.00%) -	72 (81.80%) 1000.00 (792.00:1000.00)	N/A
Oxycodone or morphine	Patients receiving Median dose (mg)	0 (0.00%) -	59 (67.00%) 15 (10:30)	N/A
Alfentanil infusion	Patients receiving Median dose (*µ*g)	0 (0.00%) -	16 (18.20%) 13,020 (8625:17164)	N/A
Methadone	Patients receiving Median dose (mg)	87 (100%) 10 (10:20)	- 0 (0.00%)	N/A
**Fluid administration**				
Crystalloid fluid	Patients receiving Volume administered (mL)	87 (100.00%) 250 (250:500)	88 (100.00%) 1000 (1000:1000)	> 0.999 < 0.001
Albumex 4%	Patients receiving Volume administered (mL)	39 (44.80%) 500 (500:1000)	5 (5.68%) 500 (500:500)	< 0.001 0.168
Albumex 20%	Patients receiving Volume administered (mL)	9 (10.30%) 100 (100:200)	10 (11.40%) 100 (100:200)	> 0.999 0.962
Patient blood returned from the CPB circuit (mL)	Patients receiving Volume administered (mL)	87 (100.00%) 750.00 [700.00:800.00]	88 (100.00%) 500.00 [500.00:762.00]	> 0.999 < 0.001
Total fluid (crystalloid, colloid, CPB circuit blood)	Volume administered (mL)	1500 [1150:1950]	1525 [1500:2288]	0.001
**Proportion of patients receiving vasoactive medications**				
Metaraminol Milrinone Ephedrine Epinephrine Norepinephrine	Patients receiving Patients receiving Patients receiving Patients receiving Patients receiving	87 (100%) 16 (18.4%) 1 (1.15%) 1 (1.15%) 15 (17.20%)	88 (100%) 14 (15.9%) 6 (6.82%) 3 (3.41%) 11 (12.50%)	> 0.999 0.693 0.118 0.621 0.403
**Proportion of patients receiving blood product**				
Red blood cell transfusion Platelets Fresh frozen plasma Cryoprecipitate Prothrombinex complex concentrate	Patients receiving Patients receiving Patients receiving Patients receiving Patients receiving	20 (23.00%) 10 (11.50%) 1 (1.15%) 0 (0.00%) 15 (17.20%)	13 (14.80%) 17 (19.30%) 7 (7.95%) 6 (6.82%) 16 (18.20%)	0.181 0.209 0.064 0.029 > 0.999
**Surgical times**				
Cardiac pulmonary bypass time (min)		133.00 [110.00:156:00]	101.00 [88.00:124.00]	< 0.001
Aortic clamp time (min)		112.00 [90.00:138.00]	80.00 [65.80:96.20]	< 0.001
Duration of surgery (min)		315.00 [295.00:360.00]	315.00 [285.00:360.00]	0.920

### Primary outcome

After adjusting for BMI, public or private hospital settings, surgical urgency, CPB times, and EuroSCORE II, patients in the fast-track group had significantly shorter total ventilation times. On average, patients in the fast-track group had a 35% reduction in adjusted total ventilation hours compared with patients in the usual care group (see Table 4).

**Table 4:** Adjusted ventilation hours using linear regression

	Log_2_ Adjusted Ventilation Hours (Coefficient (95% CI))	*P*-value
*Covariate*		
Fast-track Yes No	-0.62 (-1.07:-0.17) Reference	0.007
Type of Hospital Public Private	-0.11 (-0.64:0.41) Reference	0.669
Body mass index	0.02 (-0.01:0.05)	0.146
Category of surgery Elective Urgent Emergent	Reference 0.22 (-0.28:0.72) 0.04 (-0.42:0.50)	0.390 0.858
Cardiopulmonary bypass time (min)	0.00 (-0.00:0.01)	0.156
EuroSCORE II	0.04 (-0.06:0.15)	0.387
**Observations**: ***n*** = **164** **R**^**2**^ **= 0.098** **R** ^**2**^ **adjusted = 0.058**		

### Time to tracheal extubation

**Fig. 3:**
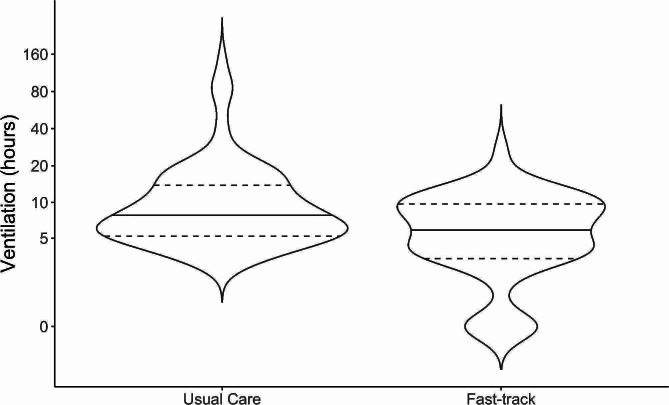
Violin plot of total mechanical ventilation time before extubation in hours between Fast-track and Usual care groups

Thirty-five patients (40.2%) in the fast-track group were extubated within the first four postoperative hours compared with 10 patients (11.4%) in the usual care group (odds ratio:5.2 [95% CI:2.39–11.08; *p* < 0.001]). The median time to extubation was 6 h (95% CI:4.0–7.5) in the fast-track group compared to 7.33 h (95% CI:6.5–9.50) in the usual care group (*p* = 0.005; see Fig. 3).

**Fig. 4:**
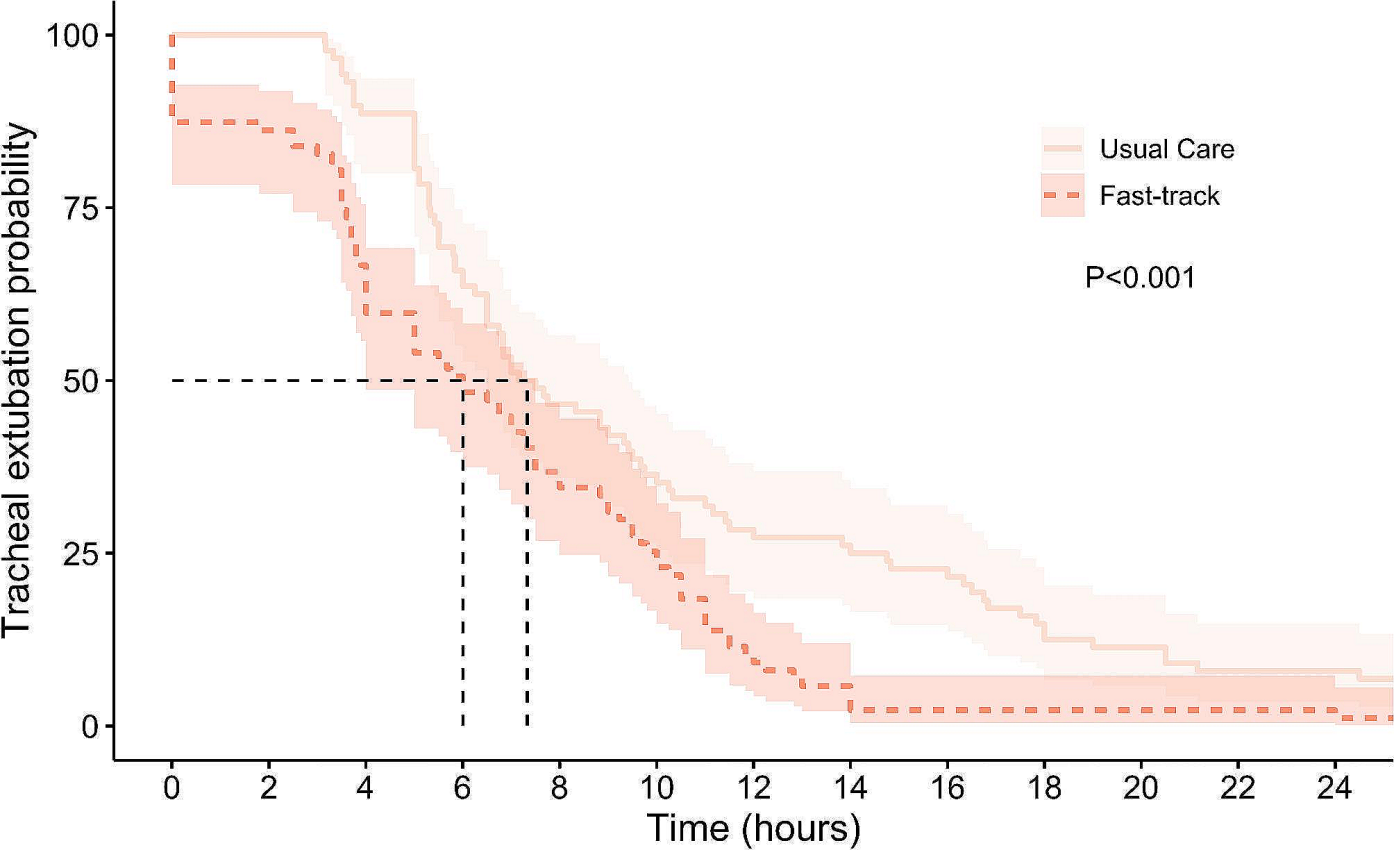
Kaplan-Meier curve showing time to tracheal extubation between Fast-track and Usual care groups with 95% confidence intervals (estimated from a log hazard). Graph is restricted to the first 24 h to allow for a better visual comparison between the groups

**Fig. 5:**
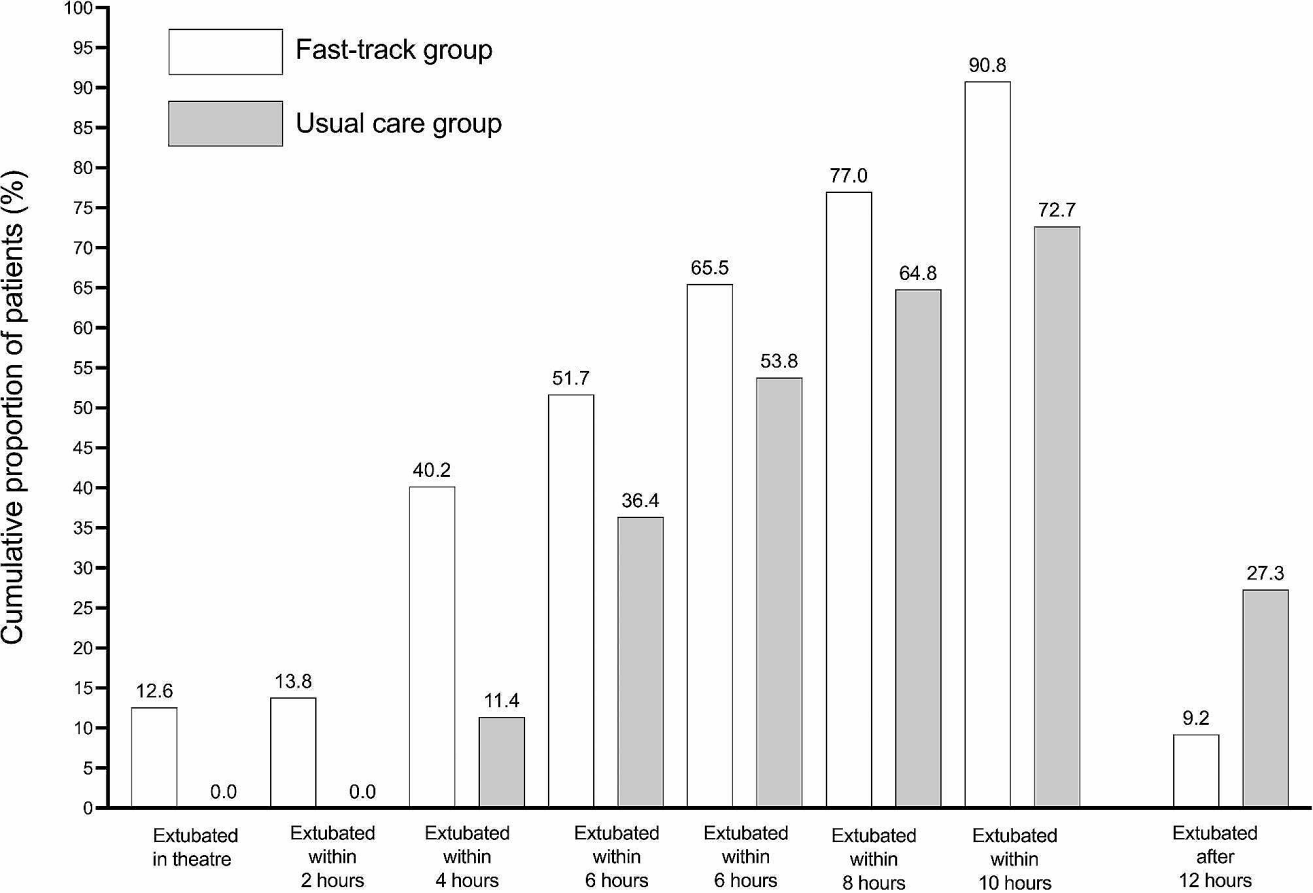
Cumulative proportion of patients and tracheal extubation times

The modified survival plot modeling “time-to-event,” where time was recorded in hours and the event was defined as tracheal extubation, is presented in Fig. 4. Eleven (12.6%) patients in the fast-track group were extubated in the operating room, compared to zero patients in the usual care group. The cumulative proportion of patients who were extubated within each two-hour period is shown in Fig. 5. Twenty-four patients (27.3%) in the usual care group remained intubated for greater than 12 postoperative hours compared to eight patients (9.2%) in the fast-track group (odds ratio:2.7 [95% CI:1.56–8.3; *p* = 0.003]).

### Pain scores and opioid use

**Fig. 6:**
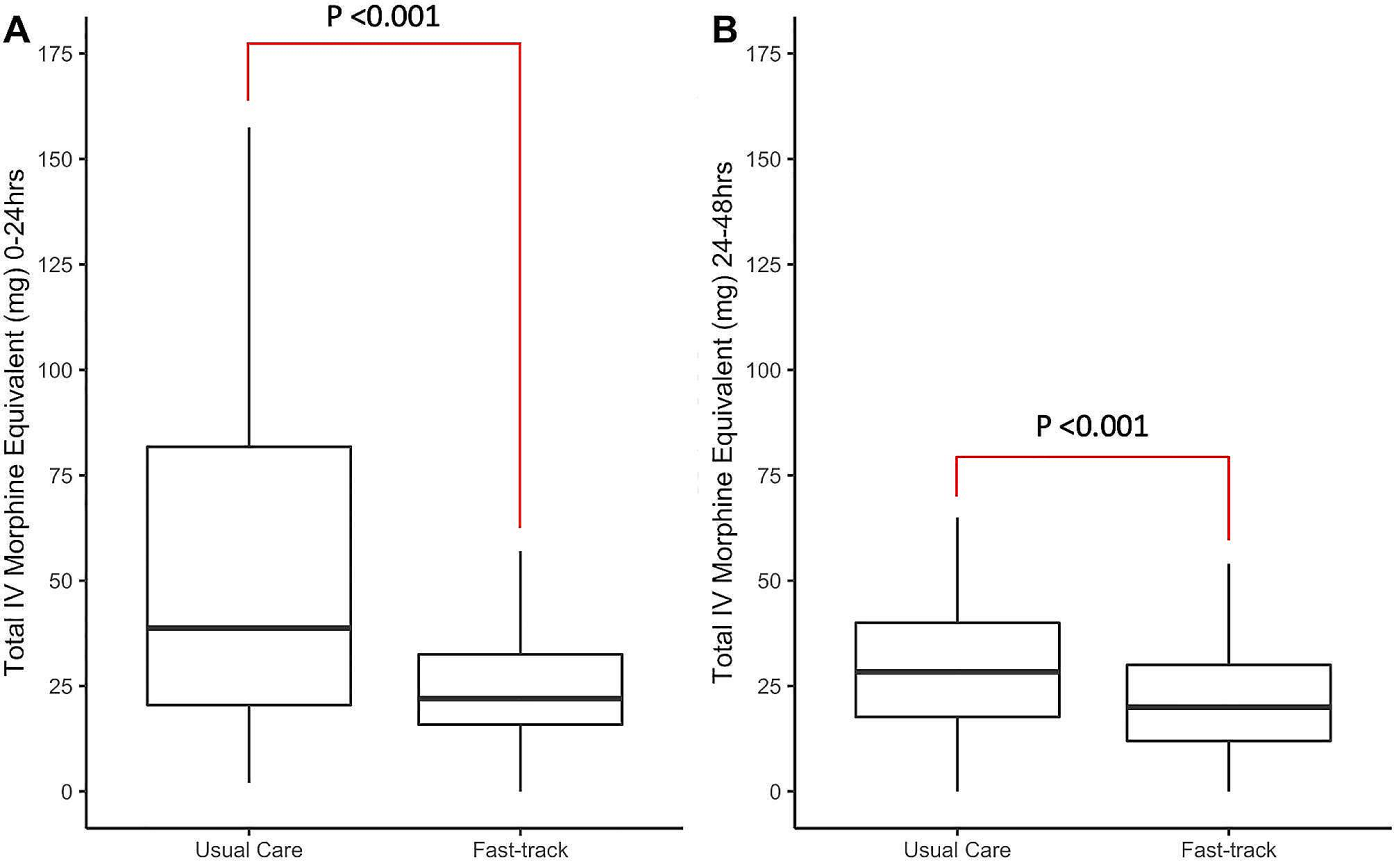
Box plots of total IV morphine equivalent use at 24 h (**A**) and 48 h (**B**) postoperatively between Fast-track and Usual care groups

Patients in the fast-track group reported less severe pain over the first 24 postoperative hours (highest pain score: 4; 95% CI:4.0–5.0) compared to 6 (95% CI:5.0–8.0) in the usual care group (*p* < 0.001). There were no significant differences in average pain scores between the groups. Patients in the Fast-track group had lower Richmond agitation scores (*p* < 0.001) and required less total IV morphine equivalent (in milligrams) compared to the usual care group at 24 h postoperatively (22.00 mg [15.75:32.50] vs. 38.75 mg [20.50:81.75]; *p* < 0.001) and 48 h postoperatively (20.00 mg [12.00:30.00] vs. 28.25 mg [17.70:40.00]; *p* < 0.001), as shown in Fig. 6.

### Complications, inpatient mortality and length of stay

None of the patients in either group developed respiratory depression requiring naloxone administration or required tracheal reintubation. Patients in the fast-track group had lower Richmond agitation sedation scores and were mobilized earlier than patients in the usual care group (Table 5). Due to different ICU discharge policies in the private hospital, the length of ICU stay was significantly longer in the fast-track group than in the usual care group: 63.00 h (44.00:86.75) vs. 45.00 h (23.00:73.75), *p* = 0.031. The length of hospital stay was shorter in the usual care group: 8.00 days [6.50:10.50] vs. 9.00 days [7.00:12.00]; however, this did not reach statistical significance (*p* = 0.402). No differences were observed in postoperative complications (Table 6).

**Table 5:** Secondary outcomes. Data are presented as a number (proportion) or a median (interquartile range)

	Fast-track (*n* = 87)	Usual care (*n* = 88)	*P*-value
Average pain score over 24 h	2.58 [2.00:4.00]	3.00 [1.74:4.67]	0.453
Highest pain score over 24 h	4.00 [3.00:5.00]	6.00 [4.00:8.00]	< 0.001
Morphine use at 24 h	22.00 [15.75:32.50]	38.75 [20.50:81.75]	< 0.001
Morphine use at 48 h	20.00 [12.00:30.00]	28.25 [17.70:40.00]	< 0.001
Richmond agitation score pre-extubation	–2 [–2:–1]	–2 [–3:–1]	< 0.001
Time to mobilize in ICU (hours)	19.90 [16.00:22.90]	22.00 [18.00:38.50]	0.007
Post-extubation arterial blood gas pH Lowest SaO_2_ (%) Lowest PaO_2_ (mmHg) Highest PaCO_2_ (mmHg) Bicarbonate (mmol/L) Standard base excess (mEq/L) Hemoglobin (g/L) Lactate (mmol/L) Potassium (mmol/L)	7.35 [7.32:7.37] 98.00 [96.20:99.00] 114.00 [88.60:152.00] 41.00 [38.10:43.90] 22.00 [21.10:23.00] –2.45 [–3.60: − 1.30] 92.00 [86.00:103.00] 1.40 [1.02:1.80] 4.40 [4.20:4.60]	7.37 [7.34:7.40] 96.00 [93.80:97.10] 80.00 [70.00:99.00] 41.00 [38.00:46.00] 24.00 [22.00:25.00] –0.10 [–1.83:1.05] 98.00 [86.75:107.00] 1.50 [1.10:2.02] 4.30 [4.10:4.50]	0.002 < 0.001 < 0.001 0.332 < 0.001 < 0.001 0.224 0.216 0.201
Length of stay – ICU (hours)	63.00 [44.00:86.75]	45.00 [23.00:73.75]	0.031
Length of stay – Hospital (days)	9.00 [7.00:12.00]	8.00 [6.50:10.50]	0.402

**Table 6:** Postoperative complications. Data are presented as number of patients (proportion) or a median (interquartile range)

	Fast-track (*n* = 87)	Usual care (*n* = 88)	*P*-value
**Bleeding and blood product use**			
Postoperative drain output (mL)	200.00 (120.00:388.00)	280.00 (120.00:550.00)	0.285
Return to theatre for bleeding	9 (10.30%)	11 (12.50%)	0.813
RBC transfusion in ICU	37 (42.50%)	33 (37.50%)	0.539
RBC units transfused in ICU	2.00 (1.00:2.00)	1.50 (1.00:2.00)	-
Fresh frozen plasma transfusion in ICU	3 (3.45%)	8 (9.09%)	0.212
Fresh frozen plasma units transfused in ICU	2.00 (2.00:2.00)	2 (1.75:3.25)	-
Platelet transfusion in ICU	3 (3.45%)	9 (10.20%)	0.132
Platelet units transfused in ICU	1.00 (1.00:1.50)	1.00 (1.00:1.00)	-
Cryoprecipitate transfusion in ICU	2 (2.30%)	5 (5.68%)	0.444
Cryoprecipitate units transfused in ICU	7.50 (6.25:8.75)	5.00 (5.00:10.00)	-
**Arrhythmias**			
Atrial fibrillation	26 (29.90%)	22 (25.00%)	0.501
Other arrhythmias requiring intervention	7 (8.05%)	7 (7.95%)	> 0.99
Need for permanent pacemaker	2 (2.30%)	5 (5.68%)	0.444
**Respiratory complications**			
Respiratory depression requiring naloxone	0 (0.00%)	0 (0.00%)	> 0.99
Pneumonia requiring antibiotics and / or high-flow oxygen	4 (4.60%)	9 (10.20%)	0.248
Tracheal reintubation in ICU	0 (0.00%)	0 (0.00%)	> 0.99
Pulmonary embolus	0 (0.00%)	0 (0.00%)	> 0.99
**Neurological complications**			
Delirium	0 (0.00%)	1 (1.14%)	> 0.99
Cerebral vascular event	1 (1.15%)	2 (2.27%)	> 0.99
Seizure	0 (0.00%)	0 (0.00%)	> 0.99
**Renal**			
Stage 1 acute kidney injury	19 (21.80%)	15 (17.00%)	0.450
Renal replacement therapy	0 (0.00%)	1 (1.14%)	0 (0.00%)
**Other**			
Surgical site infection requiring treatment	1 (1.15%)	4 (4.55%)	> 0.99
Postoperative sepsis	0 (0.00%)	0 (0.00%)	> 0.99
Pressure injury	0 (0.00%)	0 (0.00%)	> 0.99
In-hospital mortality	0 (0.00%)	1 (1.14%)	> 0.99
Unplanned readmissions - ICU	0 (0.00%)	0 (0.00%)	> 0.99
Unplanned readmissions − 30 postoperative days	0 (0.00%)	0 (0.00%)	> 0.99

## Discussion

### Key findings

In this multicentre center retrospective study, the implementation of an FTCA protocol using methadone in combination with magnesium, ketamine, lidocaine, and dexmedetomidine was associated with a significant reduction in the time to tracheal extubation, improved postoperative analgesia, and less opioid use without adverse safety events. One in ten patients in the fast-track group were extubated in the operating room versus zero patients in the usual care group. In the fast-track group over forty per cent were extubated within the first four postoperative hours, compared to 11% in the in the usual care group.

### Relationship to the literature

Our findings regarding earlier times to tracheal extubation are comparable to those of previous studies evaluating early tracheal extubation in cardiac surgery [7–9]. A meta-analysis of 28 randomized controlled trials reported that studies using low-dose opioid-based FTCA and/or a time-directed extubation protocol demonstrated a reduced time to extubation [6]. Several studies have investigated the impact of FTCA in shortening LOS in both the ICU and hospital. The use of an enhanced recovery after abdominal surgery (ERAS) protocol and FTCA significantly shortened the duration of ICU stay [9]. Similarly, other studies reported that the implementation of a dedicated ERAS protocol reduced the length of hospital stay from ten to seven days [21]. The superior analgesia observed in the FTCA group, together with a shorter mechanical ventilation time, may also explain the lower incidence of postoperative pneumonia observed in the FTCA group.

We observed no significant differences in the development of complications or hospital LOS. Other studies have reported significant benefits in these same postoperative metrics with the ERAS or FTCA program [6, 21]. Paradoxically, we found an increase in ICU LOS in the fast-track group, which reflects that more patients in this group underwent surgery in the private sector than in the public sector. These findings are concordant with the Australian and New Zealand Society of Cardiac and Thoracic Surgeons’ Cardiac Surgery Database Program, [22] which reports that ICU LOS post-CABG is longer in private hospitals than in public hospitals owing to different ward monitoring capabilities and limitations to other critical rescue services such as access to rapid response teams.

### Choices of medications to facilitate FTCA

The combination of methadone, dexmedetomidine, lidocaine, and ketamine has not been formally evaluated for FTCA. However, several lines of reasoning provide the rationale for their use. Methadone has several beneficial pharmacokinetic and pharmacodynamic properties [23, 24]. It inhibits central nervous system serotonin and norepinephrine reuptake, which may increase descending pain modulation and positively affect mood- and mood-related aspects of pain perception. Its rapid onset (approximately 4 min) and long elimination half-life (24–36 h), with stable plasma concentrations after a single intraoperative dose, make it suitable for FTCA. In addition to its strong µ-opioid receptor agonist activity, methadone is a potent N-methyl-D-aspartate (NMDA) receptor antagonist, which may attenuate the development of opioid tolerance and hyperalgesia. In cardiac surgical patients, methadone has been reported to be safe and significantly reduces intraoperative and postoperative opioid requirements [23–28].

Magnesium has been shown to improve analgesia and decrease opioid use by regulating calcium influx into the cell and antagonism of NMDA receptors in the central nervous system [29]. Dexmedetomidine is a highly selective centrally acting intravenous α_2_-receptor agonist that reduces opioid consumption and facilitates earlier discharge from hospital [30–32]. Similarly, lidocaine is an anti-inflammatory and anti-hyperalgesic agent with opioid-sparing analgesic and anti-stress effects, resulting in improvements in postoperative analgesia and enhanced recovery after surgery [33]. We chose a conservative dosing strategy using ideal body weight for lignocaine for several reasons. Commonly used drugs in cardiac surgery such as beta-adrenoreceptor antagonists and amiodarone can lower the metabolism and clearance of intravenous lignocaine during cardiac surgery increasing the risk of lignocaine toxicity. Elderly and high-risk cardiac patients, especially those with acute coronary syndrome or myocardial infarction, have modestly abnormal liver function tests. Lignocaine is metabolized by the liver; hence hepatic impairment further increases toxicity risk. Finally, lignocaine metabolism is severely abnormal in patients with cardiac dysfunction, failure, and cardiogenic shock after myocardial infarction.

Finally, ketamine is an NMDA receptor antagonist that prevents central sensitization in dorsal horn neurons. Its beneficial properties in cardiac surgery can be attributed to its analgesic, anti-hyperalgesic, and opioid-sparing effects [34, 35]. Ketamine provides additional cardiorespiratory stability and mood improvements without sedation or respiratory depression.

In a 2019 study, Markham et al. utilized IV dexmedetomidine and ropivacaine for regional anesthesia [10]. Notably, they also found that a significantly higher proportion of patients in the study group achieved extubation in the operating room, 48% (12 patients) compared to 4% (1 patient) of the control group. Dexmedetomidine has a rapid onset, achieves a peak effect within an hour of initiation, and is not associated with respiratory depression, making it an attractive option for use in FTCA. Another study showed that dexmedetomidine-based sedation resulted in shorter times to extubation than propofol-based sedation in cardiac surgery patients [36].

Our finding of improved postoperative analgesia and lower opioid use may be explained by the proven analgesic effects of each of the four agents. Methadone has a longer half-life than other opioids, resulting in a longer duration of analgesia. Methadone can reduce postoperative opioid-based analgesia requirements in cardiac surgery [37]. Its analgesic effects are synergistic with dexmedetomidine, which exerts its analgesic effect by reducing sympathetic outflow via its high affinity for α_2_ receptors, while sparing opioid receptors. Several studies have demonstrated the opioid-sparing effects of dexmedetomidine postoperatively, [30, 31] as reproduced in the present study.

### Strengths and limitations

This study has several strengths. The combined use of the above pharmacological analgesic strategy in a fast-track protocol has not been formally investigated. All statistical analyses were completed by a biostatistician who was blinded to the group allocation. The study was conducted across the public and private health sectors improving study generalizability, and electronic medical records allowed for accurate collection of granular outcome data, especially postoperative blood gas results; use of fluid, vasoactive medications, blood products; postoperative pain scores and opioid use. Finally, patient follow-up was complete, and full details of all complications, including readmissions, were collected.

This study has several limitations that are intrinsic to its retrospective design. The EuroSCORE II scores were low in all patients; therefore, the findings may not be generalizable to higher-risk CABG patients. Similarly, less than 25% of all patients underwent urgent inpatient surgery, and patients undergoing valvular cardiac surgery, redo cardiac surgery, combined CABG and valve surgery, or surgery on the aorta were excluded. The small sample size limits the systematic evaluation of clinically meaningful outcomes, such as complications and LOS. Furthermore, this study was undertaken in a well-resourced healthcare system in Australia, limiting its external validity to other regions. Healthcare costs affected by the FTCA program were not considered.

Many baseline differences in patient characteristics and cardiac investigations could not be adjusted for due to our small sample size, which may have acted as confounders. Implementing a formal protocol for FTCA across both hospitals may have introduced bias in the choice of medications or highlighted differences in practices between anesthesiologists and intensivists who cared for patients in both groups. We acknowledge that on-table extubation was feasible only for patients in the FTCA group. Key factors facilitating this included the deliberate avoidance of long-acting neuromuscular blocking agents, effective pain management, and the use of sugammadex in 100% of patients in the FTCA group. Of note, 60% of patients in the Usual care group were not administered a neuromuscular reversal agent. The strategic use of sugammadex, along with careful monitoring of neuromuscular function, played a pivotal role in achieving timely extubation and enhancing the overall efficiency of FTCA.

Lignocaine plasma levels were not measured; therefore, we are unable to assess the efficacy and safety of our lignocaine dosing strategy. We are unable to be certain as to what the specific drivers were for both the choice of fluid and the volume of fluid. The decision to administer crystalloids or colloids during cardiac surgery is multifaceted, involving a delicate balance of clinical context, echocardiographic assessments, and blood loss management. Lastly, while the retrospective design of the study limits the ability to infer causality, measures were taken to mitigate the potential impact of selection bias. Baseline characteristics were compared between groups to ensure comparability, and adjustment was made for hospital setting and relevant clinical and surgical risk factors to account for potential unmeasured confounders. Nonetheless, randomized controlled trials are needed to further validate the findings of this study.

## Conclusions

Implementing an FTCA protocol using methadone, dexmedetomidine, magnesium, ketamine, lignocaine, and remifentanil together with protocolized weaning from the mechanical protocol was associated with reduced time to tracheal extubation, reduced pain scores, and reduced postoperative opioid use without increased risks of postoperative adverse events, tracheal reintubations, or unplanned readmission to the ICU. A prospective randomized trial is warranted to further investigate the combined effects of these medications in reducing complications and LOS in FTCA.

### Electronic supplementary material

Below is the link to the electronic supplementary material.

Supplementary file 1: Definitions of complications as per standards for definitions and use of outcome measures for clinical effectiveness research in perioperative medicine

Supplementary file 2: R Packages used for statistical analysis

Supplementary file 3: De-identified database

## Data Availability

The full dataset is provided within the mansucript as a supplementary file.

## Abbreviations

AKI: Acute kidney injury
BMI: Body mass index
CABG: Coronary artery bypass graft
CI: Confidence interval
CPB: Cardiopulmonary bypass
EuroSCORE: European System for Cardiac Operative Risk Evaluation
FTCA: Fast-track cardiac anesthesia
ICU: Intensive care unit
IV: Intravenous
LOS: Length of stay
MAP: Mean arterial pressure
