# Supplementary material for: Methadone in combination with magnesium, ketamine, lidocaine, and dexmedetomidine improves postoperative outcomes after coronary artery bypass grafting: an observational multicentre study
Source: J Cardiothorac Surg. 2024 Jun 26;19:375. doi: 10.1186/s13019-024-02935-0 (PMC11202251; doi:10.1186/s13019-024-02935-0)
Supplement: Supplementary file 1 — Definitions of complications as per standards for definitions and use of outcome measures for clinical effectiveness research in perioperative medicine [file 13019_2024_2935_MOESM1_ESM.docx]

**Supplementary file Table 1.** Definitions of complications as per standards for definitions and use of outcome measures for clinical effectiveness research in perioperative medicine.^1^

| **Complication** | **Definition** | | | |
| --- | --- | --- | --- | --- |
| Postoperative pain | Numerical rating scale, where 0 was no pain, and 10 was the worst pain experienced | | | |
| Severe pain | Numerical rating pain score >6 out of 10 | | | |
| Respiratory depression | Respiratory rate <8 breaths minute requiring naloxone | | | |
| Sedation | **Richmond Agitation-Sedation Scale** | | | |
|  | **Score** | **Term** | **Description** | |
|  | +4 | Combative | Overtly combative, violent, immediate danger to staff | |
|  | +3 | Very agitated | Pulls or removes tube(s) or catheter(s); aggressive | |
|  | +2 | Agitated | Frequent non-purposeful movement, fights ventilator | |
|  | +1 | Restless | Anxious but movements not aggressive vigorous | |
|  | 0 | Alert and calm |  | |
|  | -1 | Drowsy | Not fully alert, but has sustained awakening (eye-opening/eye contact) to voice (>10 seconds) | |
|  | -2 | Light sedation | Briefly awakens with eye contact to voice (<10 seconds) | |
|  | -3 | Moderate sedation | Movement or eye opening to voice (but no eye contact) | |
|  | -4 | Deep sedation | No response to voice, but movement or eye opening to physical stimulation | |
|  | -5 | Unarousable | No response to voice or physical stimulation | |
| Pneumonia (including ventilator associated pneumonia) | The Centre for Disease Classification definition of pneumonia:  Two or more serial chest radiographs with at least one of the following (one radiograph is sufficient for patients with no underlying pulmonary or cardiac disease):  (1) new or progressive and persistent infiltrates  (2) consolidation  (3) cavitation;  at least one of the following  (1) fever (>38.8^0^C) with no other recognised cause  (2) leucopenia (white cell count <4 x10^9^/L) or leucocytosis (white cell count >12 x10^9^/L)  (3) for adults >70 years old, altered mental status with no other recognised cause;  and at least two of the following:  (1) new onset of purulent sputum or change in character of sputum, or increased respiratory secretions, or increased suctioning requirements  (2) new onset or worsening cough, or dyspnoea, or tachypnoea  (3) rales or bronchial breath sounds  (4) worsening gas exchange (hypoxemia, increased oxygen requirement, increased ventilator demand). | | | |
| Delirium | Identified using the Intensive Care Delirium Screening Checklist. Patients are first  evaluated for an altered level of consciousness. Those with a response to mild or moderate stimulation, an exaggerated response to stimulation or normal wakefulness are evaluated fully. Patients receive one point for each of the following criteria: inattention, disorientation, hallucination-delusion-psychosis, psychomotor agitation or retardation, inappropriate speech or mood, sleep/wake cycle disturbance or symptom fluctuation. Delirium is diagnosed with a score >4. | | | |
| Acute kidney injury | Kidney Disease Improving Global Outcomes (KDIGO) guidelines | | | |
|  | **Stage** | **Serum creatinine** | | **Urine output** |
|  | 1 | 1.5–1.9 times baseline value within 7 days  or  27mmol/L (0.3 mg dl/L) increase within 48 h | | < 0.5 ml kg/ hr for 6–12 h |
|  | 2 | 2.0–2.9 times baseline value within 7 days | | < 0.5 ml kg/hr for 12 h |
|  | 3 | 3.0 times baseline within 7 days  or  Increase in serum creatinine to 354 mmol/L (>4.0mg/dL) with an acute rise of > 44 mmol/L (0.5 mg/dl)  or  Initiation of renal replacement therapy | | 0.3 ml/kg/h for 24 h  or  Anuria for 12 h |
| Cardiac arrhythmias | Electrocardiograph (ECG) evidence of cardiac rhythm disturbance requiring medical intervention | | | |
| Cerebrovascular events | The American College of Surgeons National Surgical Quality Improvement Program definition: embolic, thrombotic or haemorrhagic cerebral event with persistent residual motor, sensory or cognitive dysfunction (e.g., hemiplegia, hemiparesis, aphasia, sensory deficit, impaired memory) | | | |
| Surgical site infection | **Superficial.** Defined by Centre Disease Classification as one which meets the following criteria:  (1) Infection occurs within 30 days after surgery and  (2) Involves only skin and subcutaneous tissue of the incision and  (3) The patient has at least one of the following:  (a) purulent drainage from the superficial incision  (b) organisms isolated from an aseptically obtained culture of fluid or tissue from the superficial incision  (c) at least one of the following symptoms or signs of infection: pain or tenderness, localised swelling, redness or  heat, and superficial incision is deliberately opened by surgeon and is culture positive or not cultured. A culture negative finding does not meet this criterion.  (d) diagnosis of an incisional surgical site infection by a surgeon or attending physician.  **Surgical site infection (deep).** Defined by Centre Disease Classification as one which meets the following criteria:  (1) Infection occurs within 30 days after surgery if no implant is left in place or 1 year if implant is in place.  (2) Involves deep soft tissues (e.g. fascial and muscle layers) of the incision.  (3) The patient has at least one of the following:   - purulent drainage from the deep incision but not from the organ/space component of the surgical site - a deep incision spontaneously dehisces or is deliberately opened by a surgeon and is culture-positive or not cultured when the patient has at least one of the following symptoms or signs: fever (>388C), or localised pain or tenderness. A culture-negative finding does not meet this criterion. - an abscess or other evidence of infection involving the deep incision is found on direct examination, during surgery, or by histopathological or radiological examination - diagnosis of an incisional surgical site infection by a surgeon or attending physician. | | | |

^1^ Jammer I, Wickboldt N, Sander M, Smith A, Schultz MJ, Pelosi P, Leva B, Rhodes A, Hoeft A, Walder B, Chew MS, Pearse RM; European Society of Anaesthesiology (ESA) and the European Society of Intensive Care Medicine (ESICM); European Society of Anaesthesiology; European Society of Intensive Care Medicine. Standards for definitions and use of outcome measures for clinical effectiveness research in perioperative medicine: European Perioperative Clinical Outcome (EPCO) definitions: a statement from the ESA-ESICM joint taskforce on perioperative outcome measures. Eur J Anaesthesiol. 2015 Feb;32(2):88-105. https://doi.org/10.1097/eja.0000000000000118. PMID: 25058504.
