# Supplementary material for: Methadone in combination with magnesium, ketamine, lidocaine, and dexmedetomidine improves postoperative outcomes after coronary artery bypass grafting: an observational multicentre study
Source: J Cardiothorac Surg. 2024 Jun 26;19:375. doi: 10.1186/s13019-024-02935-0 (PMC11202251; doi:10.1186/s13019-024-02935-0)
Supplement: Supplementary file 2 — R Packages used for statistical analysis [file 13019_2024_2935_MOESM2_ESM.docx]

**Supplementary file Table 2.** R Packages used for statistical analysis

|  | **R Package** |
| --- | --- |
| 1 | R Core Team (2022). R: A language and environment for statistical computing. R  Foundation for Statistical Computing, Vienna, Austria. URL <https://www.R-project.org/> |
| 2 | Wickham H, Bryan J (2022). _readxl: Read Excel Files_. R package version  1.4.0, <https://CRAN.R-project.org/package=readxl> |
| 3 | Wickham H, François R, Henry L, Müller K (2022). _dplyr: A Grammar of Data  Manipulation_. R package version 1.0.9, <https://CRAN.R-project.org/package=dplyr>.  Andri Signorell et mult. al. (2022). DescTools: Tools for descriptive  statistics. R package version 0.99.47 |
| 4 | H. Wickham. ggplot2: Elegant Graphics for Data Analysis. Springer-Verlag New  York, 2016 |
| 5 | Wilke C (2020). _cowplot: Streamlined Plot Theme and Plot Annotations for  'ggplot2'_. R package version 1.1.1,  <https://CRAN.R-project.org/package=cowplot> |
| 6 | Kassambara A (2020). _ggpubr: 'ggplot2' Based Publication Ready Plots_. R  package version 0.4.0, <https://CRAN.R-project.org/package=ggpubr> |
| 7 | Yanagida T (2022). _misty: Miscellaneous Functions 'T. Yanagida'_. R package  version 0.4.6, <https://CRAN.R-project.org/package=misty> |
| 8 | Lüdecke D (2022). _sjPlot: Data Visualization for Statistics in Social  Science_. R package version 2.8.12, <https://CRAN.R-project.org/package=sjPlot>.  Yuan Tang, Masaaki Horikoshi, and Wenxuan Li. "ggfortify: Unified Interface to  Visualize Statistical Result of Popular R Packages." The R Journal 8.2 (2016):  478-489 |
| 9 | Masaaki Horikoshi and Yuan Tang (2016). ggfortify: Data Visualization Tools  for Statistical Analysis Results. https://CRAN.R-project.org/package=ggfortify |
| 10 | Therneau T (2022). _A Package for Survival Analysis in R_. R package version  3.3-1, <https://CRAN.R-project.org/package=survival> |
| 11 | Terry M. Therneau, Patricia M. Grambsch (2000). _Modeling Survival Data:  Extending the Cox Model_. Springer, New York. ISBN 0-387-98784-3 |
| 12 | Kassambara A, Kosinski M, Biecek P (2021). _survminer: Drawing Survival Curves using 'ggplot2'_. R package version 0.4.9, <https://CRAN.R-project.org/package=survminer> |
